# O.6.1-8 Exploration of health outcomes among adolescent peer leaders of a school based physical activity intervention: baseline data

**DOI:** 10.1093/eurpub/ckad133.267

**Published:** 2023-09-11

**Authors:** Kathleen McNally, Elaine Murtagh, Kwok Ng, Catherine Woods

**Affiliations:** Physical Activity for Health Research Centre, Health Research Institute, The Department of Physical Education and Sports Science, University of Limerick, Ireland; Physical Activity for Health Research Centre, Health Research Institute, The Department of Physical Education and Sports Science, University of Limerick, Ireland; Physical Activity for Health Research Centre, Health Research Institute, The Department of Physical Education and Sports Science, University of Limerick, Ireland; School of Educational Sciences and Psychology, University of Eastern Finland; Faculty of Education, University of Turku, Finland; Kathleen.Mcnally@UL.ie; Physical Activity for Health Research Centre, Health Research Institute, The Department of Physical Education and Sports Science, University of Limerick, Ireland

## Abstract

**Purpose:**

Low physical activity (PA) levels among adolescents emphasises the need for PA interventions. School-based interventions that involve peer leaders (PL) such as the second-level Active School Flag (SLASF) program may be a useful way to encourage PA among adolescents. However, it is unclear what effect being a peer leader of a PA intervention has on PL physical, social and mental health outcomes. This study aims to explore PL health outcomes at the initial stages of peer leading a PA intervention.

**Methods:**

Quasi-experimental research design was used to test if being PL provides physical, social and mental health benefits in comparison to non-PL (control). In Autumn 2022, senior students in Irish second level schools in the second year of the SLASF program were recruited. Students completed a self-report questionnaire, consisting of moderate-vigorous PA (MVPA) levels (PACE+), wellbeing (WHO5) and social connectedness. Social connectedness was assessed using three items and grouped into *good social connectedness* (yes to all three) or *poor social connectedness.*

Data was analysed using IBM SPSS statistics. To compare PL of the SLASF program and non-PL students, a Chi-Square test of Association was used to explore social connectedness. Wellbeing and average number of days per week of MVPA was not normally distributed (p < 0.05), therefore a Mann-Whitney U Test compared groups.

**Results:**

Seven schools, 277 adolescents (63.5% male) aged 15-16 years completed the online questionnaire, 49% were in the PL group and 51% in non-PL group. At baseline, no differences existed in median wellbeing scores (Mdn=21.0) (U = 7750.500, Z= -0.953, p = 0.341) and days of MVPA per a week (Mdn=4.5) (U = 7320.500, Z= -0.105, p = 0.916) between the PL and non-PL group. Although more students in the PL group (68%) had *good social connectedness* compared to non-PL group (58%) the differences were not statistically significant (X^2^(1) > = 1.724, p = 0.189).

**Conclusions:**

At the initial stages of peers leading a PA intervention, no statistically significant differences in social and mental health outcomes were observed. Future research could examine health outcomes of PL over the course of the peer led physical activity intervention.

**Support/Funding Source:**

*Mayo Educaton Centre*.

